# Novel macrocycles – and old ones doing new tricks

**DOI:** 10.3762/bjoc.15.178

**Published:** 2019-08-01

**Authors:** Wei Jiang, Christoph A Schalley

**Affiliations:** 1 Shenzhen Grubbs Institute and Department of Chemistry, Southern University of Science and Technology, Xueyuan Boulevard 1088, Shenzhen 518055, China; 2 Institut für Chemie und Biochemie, Organische Chemie, Freie Universität Berlin, Takustraße 3, 14195 Berlin, Germany

**Keywords:** macrocycles, supramolecular chemistry

Macrocycles [1] are the workhorses in supramolecular chemistry. Many basic supramolecular concepts have been developed through studying crown ethers, cryptands, podands and spherands in the 1970s and 1980s. For these contributions, Charles Pedersen, Donald J. Cram and Jean-Marie Lehn were awarded the Nobel Prize in Chemistry in 1987. In the 80s and 90s, Jean-Pierre Sauvage and Sir Fraser Stoddart used macrocycles to realize machine-like molecular motion, and they shared the Nobel Prize in Chemistry in 2016 with Ben Feringa. Clearly, macrocycles played a central role for the fundamental science that established supramolecular chemistry as an independent field of chemical research as well as for its applications in contemporary research on functional supramolecules and materials.

Nowadays, the use of macrocycles has significantly diversified. They are not only tools for studying molecular recognition and molecular machines, but are also key components for sensing, supramolecular catalysis, (chiral) separation, drug delivery, or smart materials. Many new macrocycles have recently been reported, with pillararenes being one of the most prominent examples. These new macrocycles often possess new properties. In addition, the old ones have also been taught new tricks. Together, this enormously expands the abilities and the utility of macrocycles; they "boldly go, where no one has gone before", exploring a true supramolecular universe. This special issue in the *Beilstein Journal of Organic Chemistry* gathers world-renowned experts in the field to share their very recent results and will surely help stimulate macrocyclic chemistry.

Wei Jiang and Christoph A. Schalley

Shenzhen, Berlin, July 2019
